# Supplementary material for: Phosphoproteome profiling uncovers a key role for CDKs in TNF signaling
Source: Nat Commun. 2021 Oct 18;12:6053. doi: 10.1038/s41467-021-26289-6 (PMC8523534; doi:10.1038/s41467-021-26289-6)
Supplement: Supplementary file 1 — Supplementary Information [file 41467_2021_26289_MOESM1_ESM.pdf]

“Phosphoproteome profiling uncovers a key role for CDKs in TNF signaling”

Tanzer e*t al.*

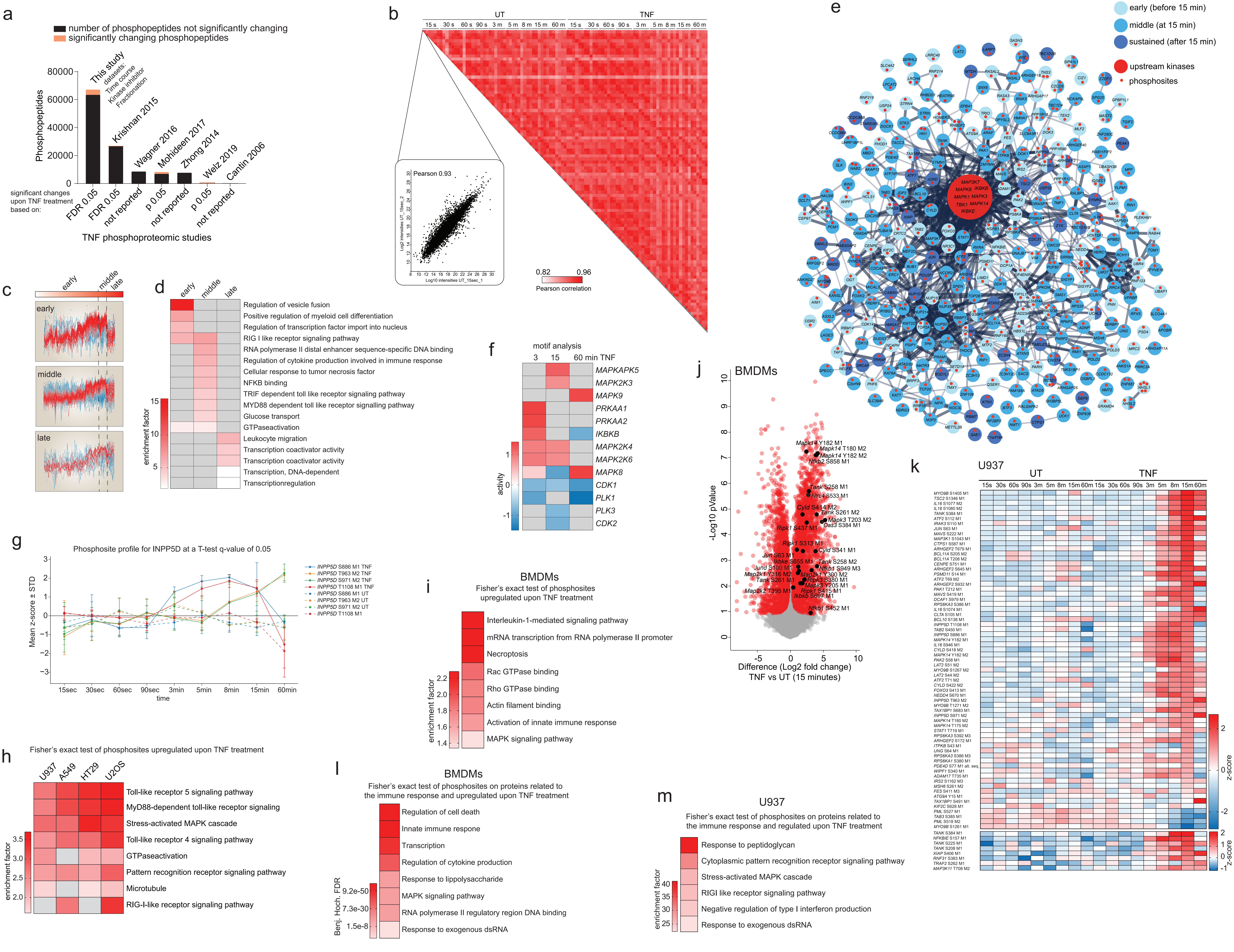

Supplementary Figure 1: Members of other immune pathways are differentially phosphorylated upon TNF treatment.
(a) Comparison of phosphopeptide numbers of previous phosphoproteome studies investigating TNF signaling with our study (Supplementary References).
(b) Phosphoproteome correlation matrix of untreated and TNF-treated samples (Pearson correlation). The scatter plot represents the correlation between two replicates.
(c) Z-scored profiles of early (significantly upregulated up to 5 min of TNF treatment compared to the respective untreated control (two-sided Student's t-test FDR < 0.05)), middle (significantly upregulated only at 15 min of TNF stimulation), and late phosphorylations (significantly upregulated only at 60 min of TNF treatment). Each profile is color coded according to its distance from the respective cluster center (red is close to center, blue is further away from center).
(d) Fisher's exact test of early, middle, and late regulated phosphosites (p < 0.01). The enrichment factor increases along the red gradient. Grey means not regulated.
(e) Network analysis (STRING) of significantly changing phosphopeptides along the time course (two-sided Student's t-test FDR < 0.05).
(f) Motif analysis (see Methods) reveals kinase activity regulation at three different time points of TNF stimulation. Red scale represents increased kinase activity, while blue represents decreased activity and grey means not regulated.
(g) Mean of z-scored intensities of phosphosites detected on INPP5D (± SD, n = 4 biologically independent experiments).
(h) Fisher's exact test of phosphosites significantly upregulated upon TNF signalling in U937, A549, HT29 and U2OS cells (p-value < 0.02). The enrichment factor increases along the red gradient. Grey means not regulated.
(i) Fisher's exact test of proteins with significantly increased phosphosites upon TNF treatment in BMDMs (p < 0.02). The following enrichment annotations are used: GOBP, GOCC, GOMF, keywords, and KEGG terms.
(j) Scatter plot of phosphosites regulated upon 15 min of TNF-treatment in BMDMs. Red dots represent significantly changing phosphosites (two-sided Student's t-test FDR < 0.05).
(k) Heatmap of means of z-scored phosphosite intensities significantly changing in U937 cells upon TNF treatment compared to the untreated control filtered for the GOBP term 'immune system process' and various other GOBP enrichment terms, which include the term' immune response' (two-sided Student's t-test FDR < 0.05).
(l) Fisher's exact test of proteins with upregulated phosphosites upon TNF stimulation filtered for the GOBP term' immune system process' and various other GOBP enrichment terms, which include the term 'immune response' (FDR < 0.02). The enrichment factor of all results was 26.2.
(m) Fisher's exact test of proteins in (k) compared to the whole dataset (FDR < 0.02).
Source data are provided as source file.

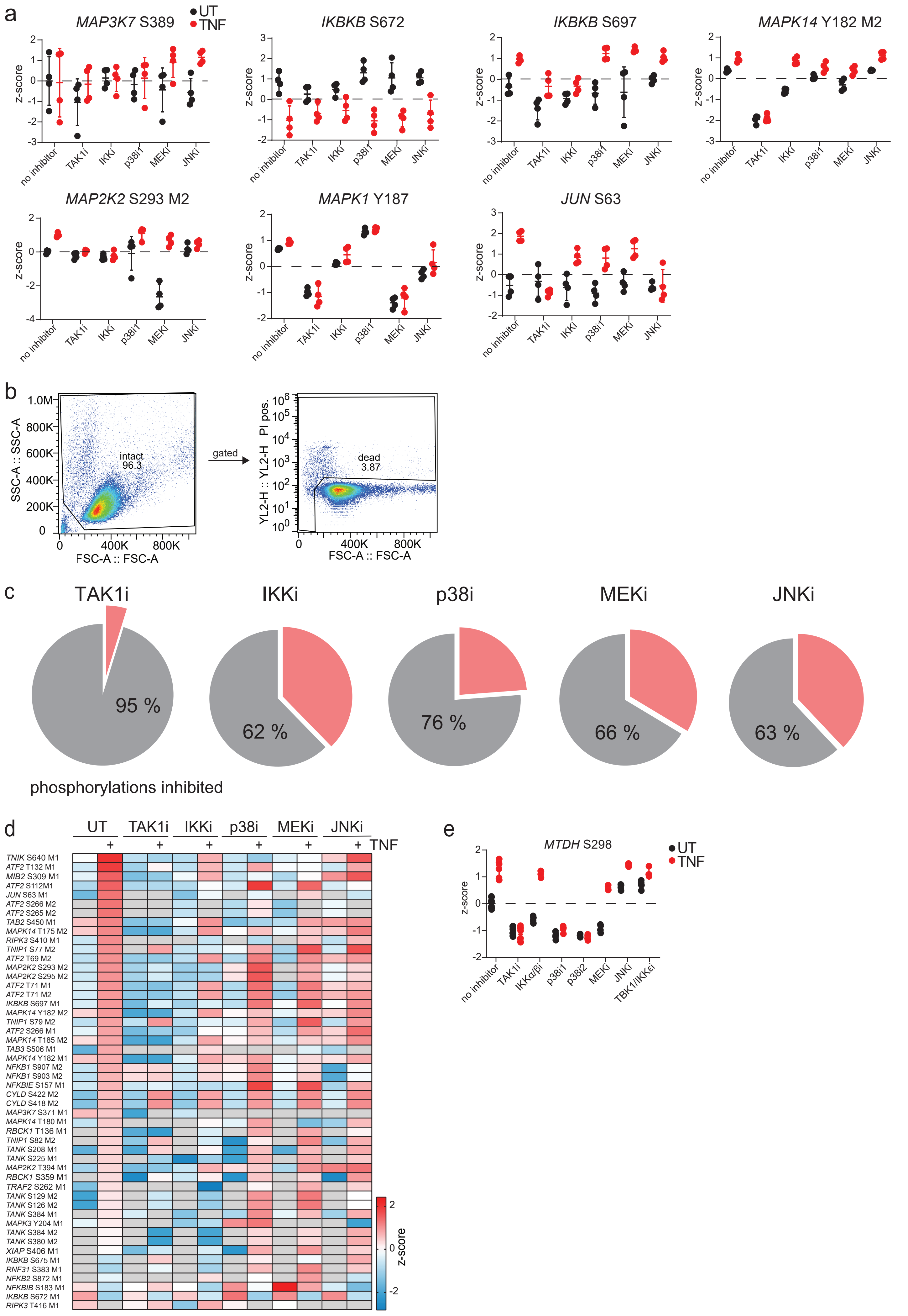

Supplementary Figure 2: Kinase inhibitors have a major impact on the TNF-phosphoproteome.
(a) Z-scored phosphosite intensities detected on known kinase substrates or kinases that are targeted by kinase inhibitors upon TNF treatment and in untreated conditions (± SD, n = 3 biologically independent experiments).
(b) Gating strategy of flow cytometry data to determine percentage of dead cells (Propidium iodide positive cells) (FlowJo 10.6.1).
(c) Pie charts of phosphosites significantly changing upon TNF treatment compared to the untreated control represent 100% (two-sided Student's t-test FDR < 0.05). Inhibition of regulated phosphosites upon TNF treatment by different kinase inhibitors is shown in grey, while phosphosites still changing (two-sided Student's t-test -Log10 p > 2.0) upon the addition of kinase inhibitors are depicted in pink.
(d) Heatmap of means of z-scored phosphosite intensities on known classical members of the TNF signaling pathway.
(e) Z-scored phosphorylation levels of S298 on MTDH in untreated (open black circles) and TNF-treated cells (red open circles) in the presence of the indicated kinase inhibitors (± SD, n = 3 biologically independent experiments).
Source data are provided as source file.

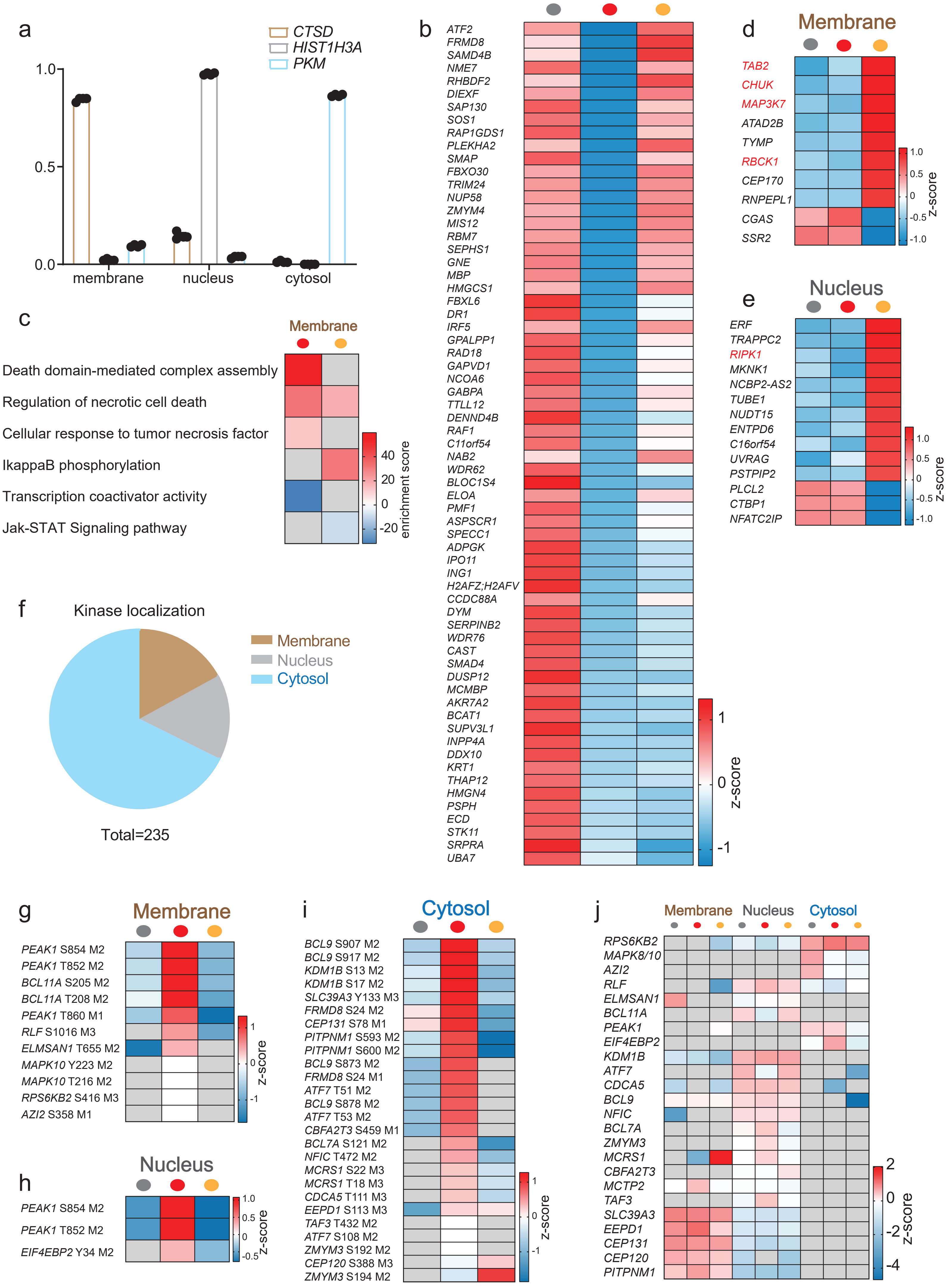

Supplementary Figure 3: Protein translocation upon TNF-treatment.
(a) Normalized profiles of marker proteins of the membrane (CTSD), nuclear (HIST1H3A), and cytosolic (PKM) fractions throughout the three different fractions (± SD, n = 4 biologically independent experiments).
(b) Heatmap of means of z-scored protein intensities de-enriched in the membrane upon TNF treatment (two-sided Student's t-test -Log10 p > 1.3, Log2 fold change < -0.5).
(c) Fisher's exact test of proteins enriched and de-enriched (as shown in Fig. 3D and Fig. S3B) in the membrane fraction ($p < 0.002$). A negative enrichment score represents proteins downregulated upon treatment.
(d) Heatmap of z-score means of proteins changing in the membrane fraction upon TAK1 inhibition compared to untreated and TNF treated cells (two-sided Student's t-test, -Log10 p > 1.3, Log2 fold change > 1.0 or < -1.0).
(e) Heatmap of means of z-scored protein intensities changing in the nucleus fraction upon TAK1 inhibition compared to untreated and TNF treated cells (two-sided Student's t-test -Log10 p > 1.3, Log2 fold change > 1.0 or < -1.0).
(f) Pie chart representing the significant enrichment of kinases in the membrane, nucleus, and cytosol fraction (two-sided Student's t-test FDR < 0.05).
(g-i) Heatmap of means of z-scored phosphosite intensities that are significantly changed in membrane (g), nucleus (h), and cytosol (i) while respective proteins were not detected in the respective fractions (total valid values < 3).
(j) Heatmap of means of z-scored protein intensities carrying phosphosites that are significantly changed in membrane (g), nucleus (h), and cytosol (i) while respective proteins were not detected in < 3 of 9 samples.
Source data are provided as source file.

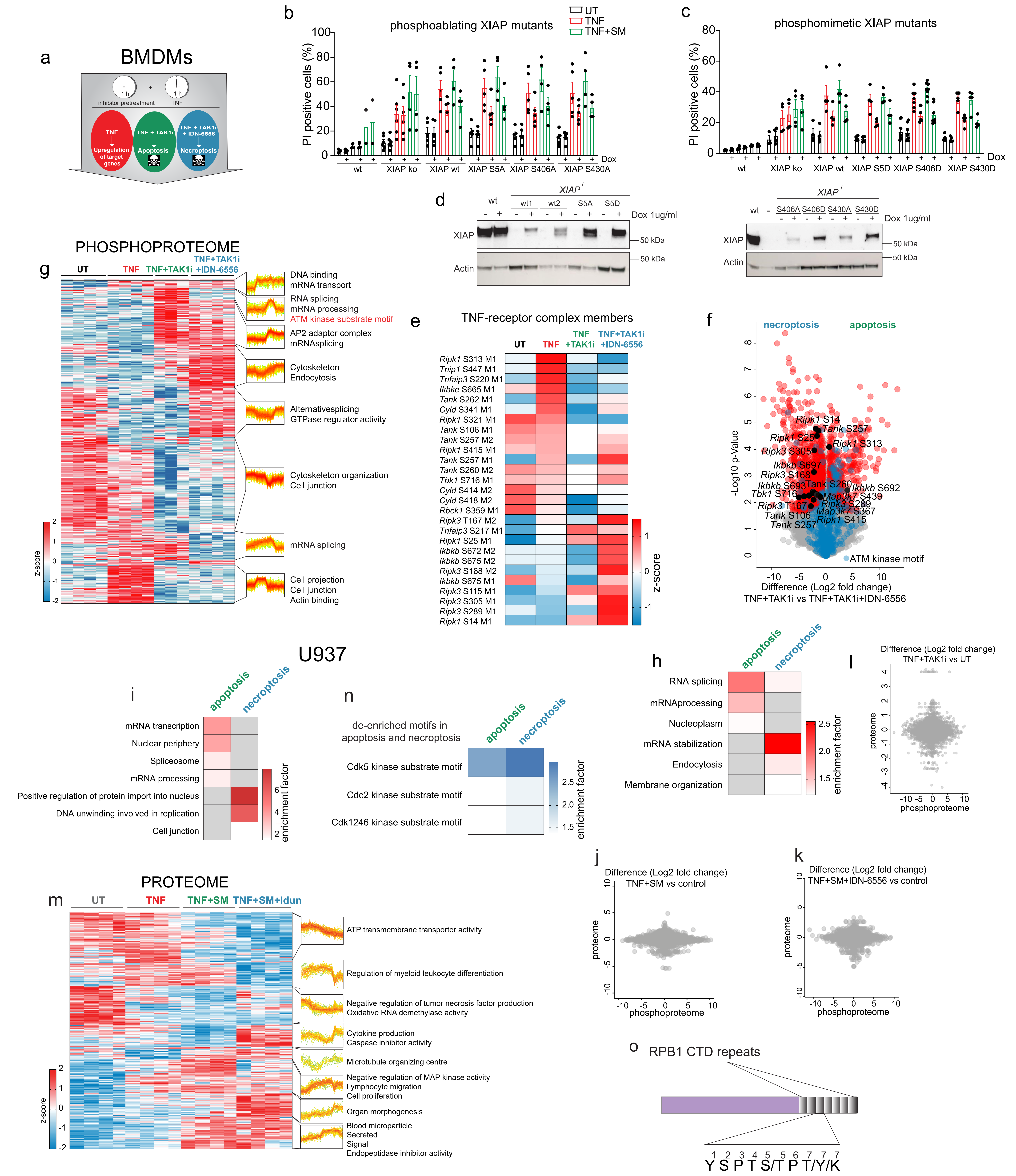

Supplementary Figure 4: The phosphoproteome of BMDMs undergoing TNF-induced apoptosis and necroptosis.
(a) Experimental scheme of BMDMs treated with TNF (100 ng/ml, red), TNF and TAK1 inhibitor (TAK1i, 7-oxozeaenol, 1 μM, green) to induce apoptosis or with TNF, TAK1i and IDN-6556 (10 μM, blue) to induce necroptosis.
(b-c) Cell death analysis of propidium iodide stained U937 wt cells and XIAP deficient cells reconstituted with the indicated phosphoablative (b) and mimetic (c) XIAP mutants that were treated with TNF alone, TNF and Smac-mimetic (SM) or left untreated. XIAP expression was induced by the addition of doxycycline (1 μg/ml) (± SD, n = 2 biologically independent experiments).
(d) Expression test of XIAP variants in U937 cells treated with doxycycline (1 μg/ml). Immunoblots were stained for XIAP and β-Actin (n =1).
(e) Heatmap of means of z-scored phosphosite intensities one-way ANOVA significantly changing and localized on proteins associated with the TNF signaling complex (FDR < 0.05).
(f) Scatter plot of phosphosites regulated upon three hours of TNF-induced apoptosis compared to TNF-induced necroptosis in BMDMs. Red phosphosites are significantly changed (two-sided Student's t-test FDR < 0.05). Blue phosphosites are part of an ATM kinase motif.
(g) Heatmap of z-scored phosphosite intensities one-way ANOVA significantly changing upon TNF, TNF-induced apoptosis, or necroptosis treatment of BMDMs for three hours (FDR < 0.05). Fisher's exact test of clusters of regulated phosphosites (FDR < 0.01). The profiles are color coded according to their distance from the respective cluster center (red is close to center, green is further away from center).
(h) Fisher's exact test of phosphosites significantly increased (FDR < 0.05) upon apoptosis and necroptosis in BMDMs (p < 0.02).
(i) Fisher's exact test of phosphosites of apoptotic and necroptotic U937 cells (p < 0.001). The red scale in (h) and (i) represents enrichment while grey shows no enrichment.
(j-l) Scatter plots of two-sided Student t-test difference comparisons of proteomes and respective phosphoproteomes of apoptotic and necroptotic U937 cells and apoptotic BMDMs.
(m) Heatmap of z-scored protein intensities one-way ANOVA significantly changing upon TNF, TNF-induced apoptosis, or necroptosis treatment for three hours in U937 cells (FDR < 0.02). Fisher's exact test of clusters of regulated proteins (p < 0.01). The profiles are color coded according to their distance from the respective cluster center (red is close to center, green is further away from center).
(n) Fisher's exact test of downregulated phosphosite motifs of apoptotic and necroptotic U937 cells (FDR < 0.02).
(o) Scheme of RPB1 and example of CTD sequence repeats. Source data are provided as source file.

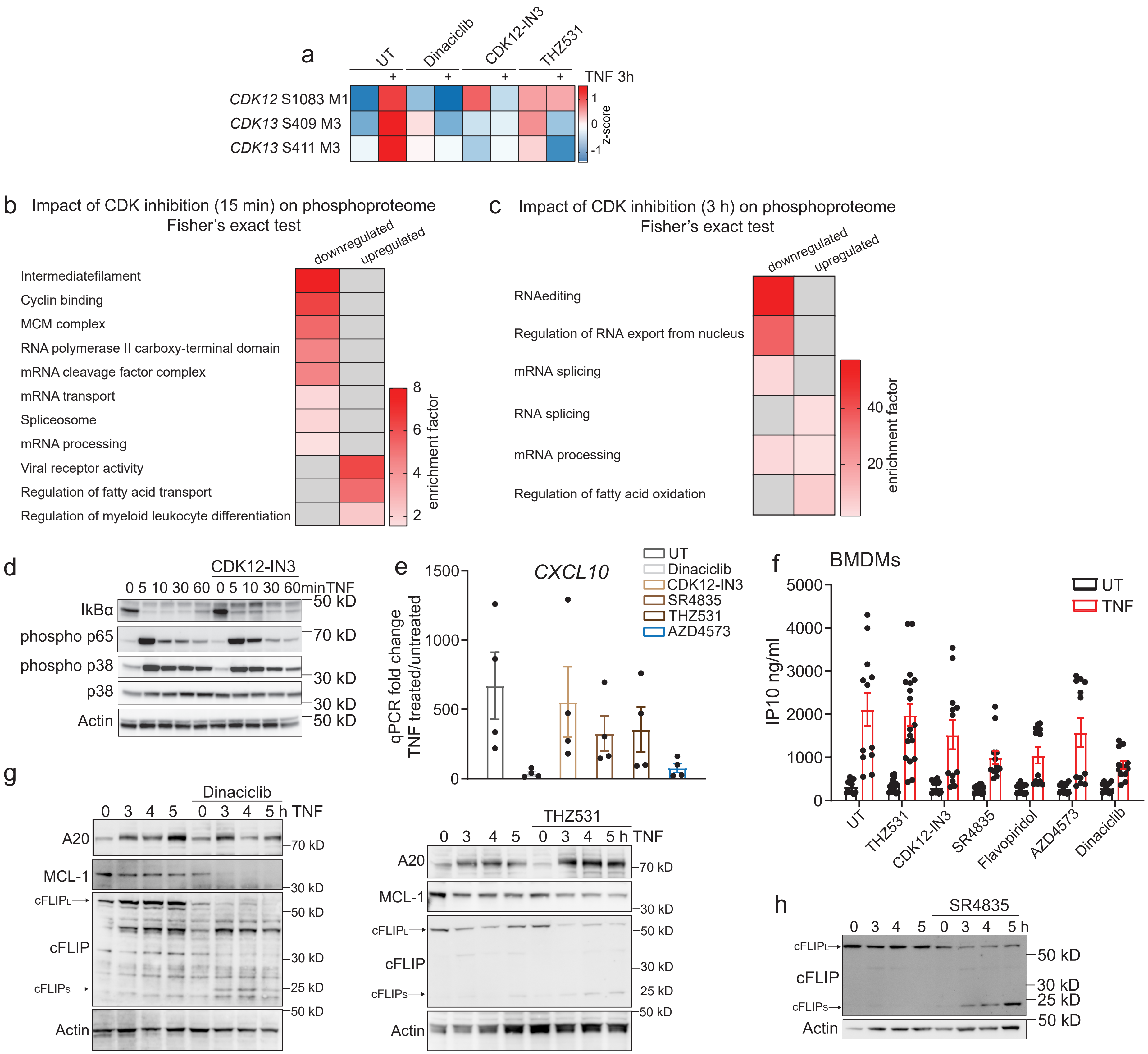

Supplementary Figure 5: Inhibition of transcriptional CDKs impacts transcription and the release of TNF-induced cytokines.
(a) Heatmap of means of z-scored phosphosite intensities on CDK12 and CDK13 significantly changing upon 3 h of TNF stimulation in combination with CDK inhibitors Dinaciclib (6 nM), CDK12-IN3 (60 nM) and THZ531 (200 nM) (two-sided Student's t-test, FDR < 0.05).
(b) Fisher's exact test of down- and upregulated phosphosites upon CDK inhibition treated as in (a) for one hour and 15 min (p < 0.02).
(c) Fisher's exact test of down- and upregulated phosphosites upon CDK inhibition by all three inhibitors tested for four hours (p < 0.02 for downregulated, p < 0.002 for upregulated).
(d) Immunoblots of U937 cells treated with TNF alone and in combination with the CDK12 inhibitor CDK12-IN3 (30 nM) stained for members of the NFκB pathway, including IκBα, phosphorylated p65 and phosphorylated and total p38 (n = 2 biologically independent experiments).
(e) qPCR of *CXCL10* (IP10) of U937 cells treated for 4 h with TNF alone or in combination with CDK inhibitors Dinaciclib (6 nM), CDK12-IN3 (60 nM), SR4835 (160 nM), THZ531 (200 nM) and AZD4573 (6 nM) (± SEM, n = 4 biologically independent experiments).
(f) ELISA of IP10 in the supernatant of BMDMs treated with or without TNF for 24 h, with and without CDK inhibitors (Dinaciclib (24 nM); Flavopiridol (60 nM); AZD5438 (24 nM); CDK12-IN3 (120 nM); SR4835 (320 nM); THZ531 (800 nM)) (± SEM, n = 6 biologically independent samples and experiments).
(g) Immunoblots of U937 cells treated with TNF alone or in combination with the panCDK inhibitor Dinaciclib (6 nM) or the CDK12/13 inhibitor THZ531 (200 nM) stained for A20, MCL1 and β-Actin levels (n = 2 biologically independent experiments).
(h) Immunoblot of U937 cells treated with TNF alone and in combination with CDK12/13 inhibitor SR4835 (160 nM) was stained for cFLIP and β-Actin. Source data are provided as source file (n = 2 biologically independent experiments).

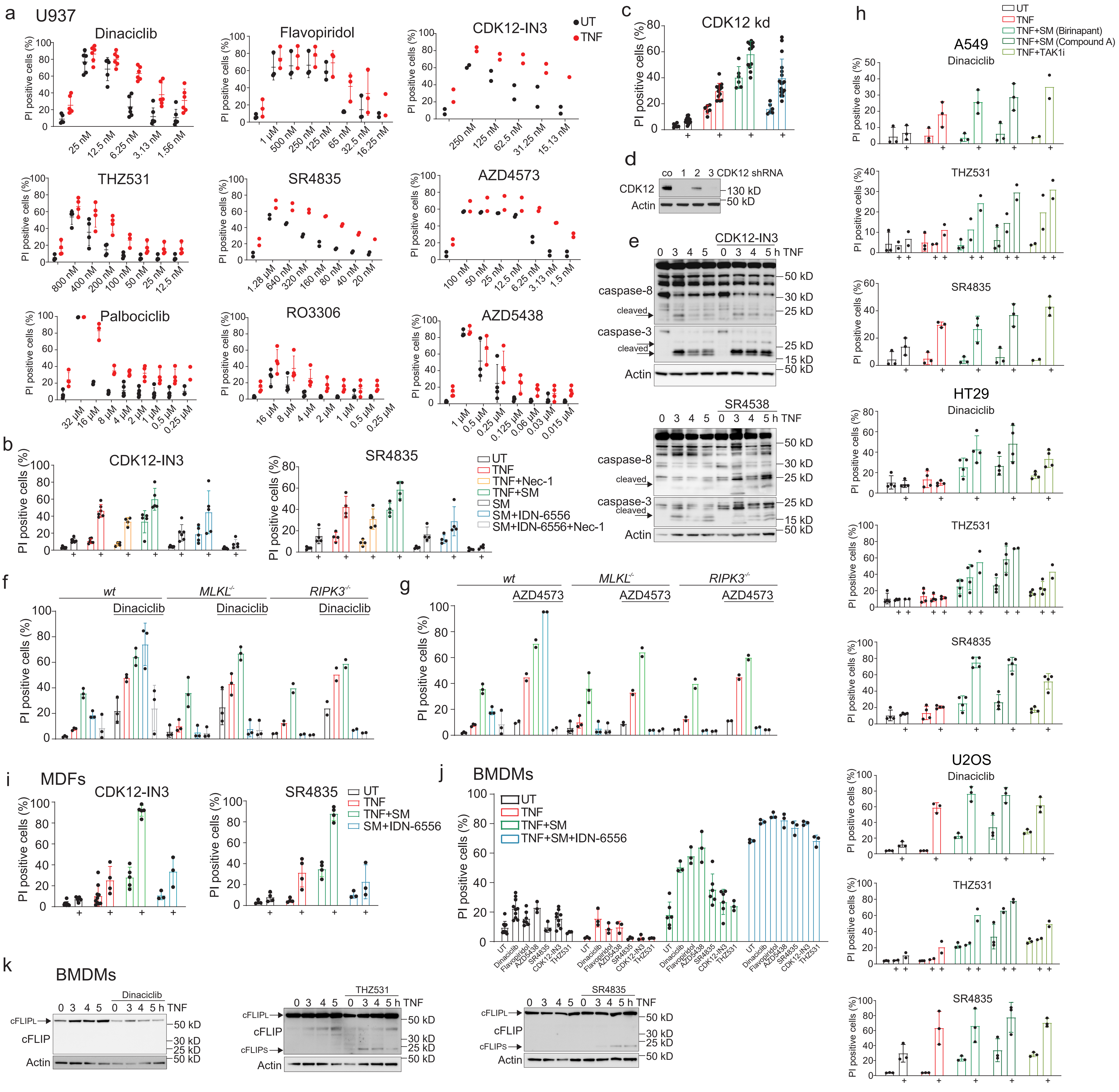

Supplementary Figure 6: Various CDK inhibitors strongly enhance TNF-induced apoptosis and necroptosis.
(a) Cell death analysis of propidium iodide stained U937 cells treated as indicated over a concentration course of various CDK inhibitors for 24 h (± SD, n = 2 biologically independent experiments).
(b) Cell death analysis by flow cytometry of propidium iodide-stained U937 cells treated as indicated (CDK12-IN3 (60 nM); SR4835 (160 nM)) for 24 h (± SD, n = 4 biologically independent experiments).
(c) Cell death analysis by flow cytometry of propidium iodide stained U937 wt or CDK12 knocked down cells stimulated with TNF, TNF, and SM or TNF, SM, and IDN-6556 for 24 h (± SD, n = 6 biologically independent experiments).
(d) Immunoblot of U937 wt and CDK12 knockdown cells stained for CDK12 and β-Actin (n = 1).
(e) Immunoblot of U937 cells stimulated with TNF alone for 3-5 h or in combination with CDK12-IN3 (60 nM) or SR4835 (160 nM) inhibitors and stained for caspase-3 and 8 and β-Actin (n = 2 biologically independent experiments).
(f-g) Cell death analysis of propidium iodide stained U937 wt cells or deficient for MLKL or RIPK3 treated as indicated (Dinaciclib (6 nM); AZD4573 (6 nM)) for 24 h (± SD, n = 2 biologically independent experiments).
(h) Cell death analysis of propidium iodide stained A549 cells, HT29 cells and U2OS cells treated as indicated (TNF (100 ng/ml); SM, Birinapant (5 µM) or Compound A (1 µM); TAK1 inhibitor 7-Oxozeaenol (1 µM)) for 24 h (± SD, n = 2 biologically independent experiments).
(i) Cell death analysis of propidium iodide stained MDFs treated as indicated (TNF (100 ng/ml); SM, Compound A (2 µM); IDN-6556 (10 µM)) for 24 h (± SD, n = 3 biologically independent samples and experiments).
(j) Cell death analysis of propidium iodide stained BMDMs treated as indicated (TNF (100 ng/ml); SM, Compound A (2 µM); IDN-6556 (10 µM); Dinaciclib (24 nM); Flavopiridol (60 nM); AZD5438 (24 nM); CDK12-IN3 (120 nM); SR4835 (320 nM); THZ531 (800 nM)) for 7 h (± SD, n = 3 biologically independent samples and experiments).
(k) Immunoblots of BMDMs treated with TNF alone and in combination with CDK inhibitors (THZ531 (800 nM); SR4835 (320 nM)) were stained for cFLIP and β-Actin (n = 2 biologically independent samples and experiments). Source data are provided as source file.

Supplementary Table 1: List of all primers and oligonucleotides used, including the names and sequences.

**Name of Oligos**	**Nucleotide sequence**
XIAP CRISPR sgRNA1	TTCTCTTTTTAGAAAAGG
XIAP CRISPR sgRNA2	GACTTTTAACAGTTTTGA
MiSeq XIAP primer fwd	ACACTCTTTCCCTACACGACGctcttccgatctACCTGCAGACATCAATAAGG AAG
MiSeq XIAP primer fwd	TGACTGGAGTTCAGACGTGTGctcttccgatctGATGGCCTGTCTAAGGCAA AATG
CDK12 shRNA1	CCGGGCTCGGCTCTATAACTCTGAACTCGAGTTCAGAGTTATAGAGCC GAGCTTTTT
CDK12 shRNA2	CCGGGCACTGAAAGAGGAGATTGTTCTCGAGAACAATCTCCTCTTTCA GTGCTTTTT
CDK12 shRNA3	CCGGGATCGATGAAGGACCGGATATCTCGAGATATCCGGTCCTTCATC GATCTTTTTG
CCL2 fwd qPCR primer	CCTAGGAATCTGCCTGATAATCGA
CCL2 rev qPCR primer	TGGGATATACCATGCATACTGAGATG
CXCL10 fwd qPCR primer	GAAATTATTCCTGCAAGCCAATTT
CXCL10 rev qPCR primer	TCACCCTTCTTTTTCATTGTAGCA
GAPDH fwd qPCR primer	GTCTCCTCTGACTTCAACAGCG
GAPDH rev qPCR primer	ACCACCCTGTTGCTGTAGCCAA

Supplementary References

1. Krishnan RK, Nolte H, Sun T, Kaur H, Sreenivasan K, Looso M, et al. Quantitative analysis of the TNF-alpha-induced phosphoproteome reveals AEG-1/MTDH/LYRIC as an IKKbeta substrate. Nat Commun. 2015;6:6658.
2. Wagner SA, Satpathy S, Beli P, Choudhary C. SPATA2 links CYLD to the TNF-alpha receptor signaling complex and modulates the receptor signaling outcomes. EMBO J. 2016;35(17):1868-84.
3. Mohideen F, Paulo JA, Ordureau A, Gygi SP, Harper JW. Quantitative Phospho-proteomic Analysis of TNFalpha/NFkappaB Signaling Reveals a Role for RIPK1 Phosphorylation in Suppressing Necrotic Cell Death. Mol Cell Proteomics. 2017;16(7):1200-16.
4. Zhong CQ, Li Y, Yang D, Zhang N, Xu X, Wu Y, et al. Quantitative phosphoproteomic analysis of RIP3-dependent protein phosphorylation in the course of TNF-induced necroptosis. Proteomics. 2014;14(6):713-24.
5. Welz B, Bikker R, Junemann J, Christmann M, Neumann K, Weber M, et al. Proteome and Phosphoproteome Analysis in TNF Long Term-Exposed Primary Human Monocytes. Int J Mol Sci. 2019;20(5).
6. Cantin GT, Venable JD, Cociorva D, Yates JR, 3rd. Quantitative phosphoproteomic analysis of the tumor necrosis factor pathway. J Proteome Res. 2006;5(1):127-34.
