# Supplementary material for: Phosphoproteome profiling uncovers a key role for CDKs in TNF signaling
Source: Nat Commun. 2021 Oct 18;12:6053. doi: 10.1038/s41467-021-26289-6 (PMC8523534; doi:10.1038/s41467-021-26289-6)
Supplement: Supplementary file 3 — Description of Additional Supplementary Files [file 41467_2021_26289_MOESM3_ESM.docx]

File Name: Supplementary Data 1:

Description: Phosphoproteome analysis of TNF-treated U937, A549, HT29 and U2OS cells. Cells were treated with TNF for 15 min. This file reports the PTM_collapse_key, which is constituted by the gene name, the position of the phosphorylation within the protein and the multiplicity, which informs about the presence of further phosphorylations on the same peptide. The UniprotID column reports the uniport IDs of proteins, which harbor the modified peptides, obtained from TrEMBL and Swissprot. Fold changes and the significance are also reported (q-value, two-sided Student’s t-test).

File Name: Supplementary Data 2:

Description: Phosphoproteome analysis of TNF-treated BMDMs. BMDMs were treated with TNF for 15 min. This file reports similar to Supplementary Data 1 phosphosites, their fold changes upon TNF stimulation and the significance (q-value, two-sided Student’s t-test).
