# Supplementary material for: Phosphoproteome profiling uncovers a key role for CDKs in TNF signaling
Source: Nat Commun. 2021 Oct 18;12:6053. doi: 10.1038/s41467-021-26289-6 (PMC8523534; doi:10.1038/s41467-021-26289-6)
Supplement: Supplementary file 6 — Reporting Summary [file 41467_2021_26289_MOESM6_ESM.pdf]

nature research

Corresponding author(s): Matthias Mann
Maria Tanzer

Last updated by author(s): Sep 29, 2021

Reporting Summary

Nature Research wishes to improve the reproducibility of the work that we publish. This form provides structure for consistency and transparency in reporting. For further information on Nature Research policies, see our Editorial Policies and the Editorial Policy Checklist.

Statistics

For all statistical analyses, confirm that the following items are present in the figure legend, table legend, main text, or Methods section.

n/a	Confirmed	
☐	☒	The exact sample size (*n*) for each experimental group/condition, given as a discrete number and unit of measurement
☐	☒	A statement on whether measurements were taken from distinct samples or whether the same sample was measured repeatedly
☐	☒	The statistical test(s) used AND whether they are one- or two-sided<br>*Only common tests should be described solely by name; describe more complex techniques in the Methods section.*
☒	☐	A description of all covariates tested
☐	☒	A description of any assumptions or corrections, such as tests of normality and adjustment for multiple comparisons
☐	☒	A full description of the statistical parameters including central tendency (e.g. means) or other basic estimates (e.g. regression coefficient) AND variation (e.g. standard deviation) or associated estimates of uncertainty (e.g. confidence intervals)
☐	☒	For null hypothesis testing, the test statistic (e.g. *F*, *t*, *r*) with confidence intervals, effect sizes, degrees of freedom and *P* value noted<br>*Give P values as exact values whenever suitable.*
☒	☐	For Bayesian analysis, information on the choice of priors and Markov chain Monte Carlo settings
☒	☐	For hierarchical and complex designs, identification of the appropriate level for tests and full reporting of outcomes
☐	☒	Estimates of effect sizes (e.g. Cohen's *d*, Pearson's *r*), indicating how they were calculated

Our web collection on statistics for biologists contains articles on many of the points above.

Software and code

Policy information about availability of computer code

Data collection	XCalibur Thermo Scientific https://www.thermofisher.com/order/catalog/product/OPTON-30487<br>Attune NxT software https://www.thermofisher.com/de/de/home/life-science/cell-analysis/flow-cytometry/flow-cytometers/attune-nxt-flow-cytometer/resources.html
Data analysis	MaxQuant (Cox and Mann, 2008), Version 1.5.0.38 http://www.biochem.mpg.de/5111795/maxquant<br>Spectronaut (Bruderer, 2015), Version 13 https://biognosys.com/shop/spectronaut<br>Perseus (Tyanova et al., 2016), Version 1.6.2.2. http://www.biochem.mpg.de/5111810/perseus<br>Prism Graphpad 8.4.3 https://www.graphpad.com/scientific-software/prism/<br>FlowJo 10.6.1 https://www.flowjo.com/<br>String (Jensen et al., 2009) http://string-db.org/<br>Python 3.7.7

For manuscripts utilizing custom algorithms or software that are central to the research but not yet described in published literature, software must be made available to editors and reviewers. We strongly encourage code deposition in a community repository (e.g. GitHub). See the Nature Research guidelines for submitting code & software for further information.

Data

Policy information about availability of data

All manuscripts must include a data availability statement. This statement should provide the following information, where applicable:

- Accession codes, unique identifiers, or web links for publicly available datasets
- A list of figures that have associated raw data
- A description of any restrictions on data availability

The MS-based proteomics data have been deposited to the ProteomeXchange Consortium via the PRIDE partner repository and are available via ProteomeXchange with identifier (PXD021366) (Jones et al., 2008).

Field-specific reporting

Please select the one below that is the best fit for your research. If you are not sure, read the appropriate sections before making your selection.

☒ Life sciences ☐ Behavioural & social sciences ☐ Ecological, evolutionary & environmental sciences

For a reference copy of the document with all sections, see nature.com/documents/nr-reporting-summary-flat.pdf

Life sciences study design

All studies must disclose on these points even when the disclosure is negative.

Sample size	No sample size collection was performed. Sample numbers are stated in each figure legend. For the DIA library samples are measured in singlicates as we aimed to identify and not quantify phosphopeptides. For DIA and DDA experiment we at least used three or more replicates per condition to allow statistical analysis.
Data exclusions	No data was excluded, all attempts of replications were successful.
Replication	For MS measurements at least 3 replicates were used. Western blot experiments were at least repeated twice and qPCR, ELISA and cell death experiments were at least repeated three times. Replicates represent biologically independent experiments and experiments with MDFs and BMDMs were additionally performed with biologically independent samples.
Randomization	The order of MS acquisitions of the time course experiment was randomized. MS acquisitions of other, smaller experiments were performed in an order specific to each respective study design e.g. replicates of the same conditions were measured together, to ensure optimal MS performance and comparability.
Blinding	Blinding was not carried out. Blinding was not relevant because the samples were all treated the same, including MS sample analysis. Flow cytometry was performed with fixed gates for all samples within an experiment.

Reporting for specific materials, systems and methods

We require information from authors about some types of materials, experimental systems and methods used in many studies. Here, indicate whether each material, system or method listed is relevant to your study. If you are not sure if a list item applies to your research, read the appropriate section before selecting a response.

Materials & experimental systems

n/a	Involved in the study	
☐	☒	Antibodies
☐	☒	Eukaryotic cell lines
☒	☐	Palaeontology and archaeology
☐	☒	Animals and other organisms
☒	☐	Human research participants
☒	☐	Clinical data
☒	☐	Dual use research of concern

Methods

n/a	Involved in the study	
☒	☐	ChIP-seq
☐	☒	Flow cytometry
☒	☐	MRI-based neuroimaging

Antibodies

Antibodies used	anti-human caspase-8 (MBL, M058-3), anti-cleaved human caspase-3 (Cell Signaling Technology CST, 9661), anti-human MLKL (Merck Millipore, MABC604), phospho anti-human RPB1 S2 (Millipore, 04-1571), anti-human RPB1 (CST, D8L4Y), phospho anti-human p65 (CST, 3033P), anti-human IκBα (CST, 9242), phospho anti-human p38 (CST, 9215), anti-human p38 (CST, 9212), anti-human CDK12 (CST, 11973), phospho anti-human CDK9 (CST, 2549), anti-human CDK9 (CST, 2316), anti-human A20 (Santa Cruz, sc-166692), anti-human MCL1 (CST,4572), anti-human XIAP (MBL, M044-3), anti-human FLIP (CST, 56343) and anti-human β-Actin (CST, 4967)

Validation	anti-human caspase-8 https://www.mblbio.com/bio/g/dtl/A/index.html?pcd=M058-3<br>anti-cleaved human caspase-3 https://www.cellsignal.com/products/primary-antibodies/cleaved-caspase-3-asp175-antibody/9661<br>anti-human MLKL https://www.merckmillipore.com/DE/de/product/Anti-MLKL-Antibody-clone-3H1,MM_NF-MABC604<br>phospho anti-human RPB1 S2 https://www.merckmillipore.com/DE/de/product/Anti-RNA-polymerase-II-subunit-B1-phospho-CTD-Ser-2-clone-3E10-rat-monoclonal.,MM_NF-04-1571-I<br>anti-human RPB1 https://www.cellsignal.com/products/primary-antibodies/rpb1-ntd-d8l4y-rabbit-mab/14958<br>phospho anti-human p65 https://www.cellsignal.com/products/primary-antibodies/phospho-nf-kb-p65-ser536-93h1-rabbit-mab/3033<br>anti-human IκBα https://www.cellsignal.com/products/primary-antibodies/ikba-antibody/9242<br>phospho anti-human p38 https://www.cellsignal.com/products/primary-antibodies/phospho-p38-mapk-thr180-tyr182-3d7-rabbit-mab/9215<br>anti-human p38 https://www.cellsignal.com/products/primary-antibodies/p38-mapk-antibody/9212<br>anti-human CDK12 https://www.cellsignal.com/products/primary-antibodies/cdk12-antibody/11973<br>phospho anti-human CDK9 https://www.cellsignal.com/products/primary-antibodies/phospho-cdk9-thr186-antibody/2549<br>anti-human CDK9 https://www.cellsignal.com/products/primary-antibodies/cdk9-c12f7-rabbit-mab/2316<br>anti-human A20 https://www.scbt.com/de/p/a20-antibody-a-12<br>anti-human MCL1 https://www.cellsignal.com/products/primary-antibodies/mcl-1-antibody/4572<br>anti-human XIAP https://www.mblintl.com/products/m044-3/<br>anti-human FLIP https://www.cellsignal.com/products/primary-antibodies/flip-d5j1e-rabbit-mab/56343<br>anti-human β-Actin https://www.scbt.com/p/actin-antibody-c-2?gclid=EAIaIQobChMIm-ieu6Tl8gIVCNZ3Ch355giFEAAYASAAEgIc6fD_BwE

Eukaryotic cell lines

Policy information about cell lines

Cell line source(s)	BMDMs and MDFs were derived by the authors themselves from mice generated by the Max-Planck Institute of Biochemistry (MPIB). U937, HT29, U2OS, A549 were received from ATCC.
Authentication	The cell lines were not authenticated.
Mycoplasma contamination	All cell lines were regularly tested negative for Mycoplasma.
Commonly misidentified lines (See ICLAC register)	No commonly misidentified cell lines were used in this study.

Animals and other organisms

Policy information about studies involving animals; ARRIVE guidelines recommended for reporting animal research

Laboratory animals	BMDMs and MDFs were derived from wildtype male mice, which were sacrificed at the age of 12 weeks according the guidelines. Mice were housed with an air-exchange rate about 15 times per hour. High-efficiency particulate air (HEPA) filters are used. An artificial light:dark cycle of 14:10 hours was set. Room temperature was set to 22 ± 1 °C and humidity conditions to 55 ± 5%. Autoclaved commercial standard diet and autoclaved water were provided.
Wild animals	No wild animals were used for this study.
Field-collected samples	No field collected samples were used for this study.
Ethics oversight	The MPIB ethics committee approved the sacrifice of the mice.

Note that full information on the approval of the study protocol must also be provided in the manuscript.

Flow Cytometry

Plots

Confirm that:

- [x] The axis labels state the marker and fluorochrome used (e.g. CD4-FITC).
- [x] The axis scales are clearly visible. Include numbers along axes only for bottom left plot of group (a 'group' is an analysis of identical markers).
- [x] All plots are contour plots with outliers or pseudocolor plots.
- [x] A numerical value for number of cells or percentage (with statistics) is provided.

Methodology

Sample preparation	U937, A549, HT29, U2OS, BMDMs and MDFs were washed with PBS and stained with Propidium iodide (PI) in PBS. The gating strategy was as published in: Murphy, J. M. et al. The pseudokinase MLKL mediates necroptosis via a molecular switch mechanism.

	Immunity, 2013 Sep 19;39(3):443-53.doi: 10.1016/j.immuni.2013.06.018. Epub 2013 Sep 5.
Instrument	Attune NxT
Software	FlowJo 10.6.1
Cell population abundance	We always analyzed one cell line at the time and did not have a mixture of cells.
Gating strategy	Cell debris with very low FSC and SSC values was excluded and the percentage of PI positive cells was determined.

☒ Tick this box to confirm that a figure exemplifying the gating strategy is provided in the Supplementary Information.
